# Chaperone-mediated remodelling of mutant huntingtin

**DOI:** 10.1098/rstb.2025.0269

**Published:** 2026-10-01

**Authors:** Kunal Gharat, Janine Kirstein

**Affiliations:** ^1^ Leibniz Institute on Aging - Fritz Lipmann Institute, Jena, Germany; ^2^ Institute of Biochemistry and Biophysics, Friedrich Schiller University, Jena, Germany

**Keywords:** chaperones, huntingtin, proteostasis

## Abstract

Huntington's disease is caused by the trinucleotide repeat CAG expansion in the huntingtin gene, resulting in an N-terminal polyglutamine (polyQ) tract that renders the huntingtin protein (HTT) prone to misfolding and aggregation. Although polyQ expansion is an intrinsic driver of aggregation, the folding trajectory of HTT is strongly shaped by molecular chaperones that detect all conformational entities of HTT and as such bind to soluble HTT and suppress *β*-sheet formation, target HTT for proteasomal degradation and resolubilize HTT fibrils by disaggregation. This review summarizes recent *in vitro* and *in vivo* data on the role of molecular chaperones in the HTTExon1 folding landscape and its cooperation with the proteolytic pathways.

This article is part of the Theo Murphy meeting issue ‘ProteostaSys: a systems view of proteostasis'.

## 1. Introduction

Huntingtin's disease is a polyglutamine (polyQ) disease. Huntington's disease (HD) is a monogenic, hereditary neurodegenerative disorder driven by an expanded glutamine (*Q*, CAG) tract in the first exon of the huntingtin (*HTT*) gene [1]. HD belongs to a group of nine polyQ diseases, all of which arise from pathogenic elongation of a polyQ segment. Although each disorder is caused by a similar type of expansion, the affected genes and associated proteins differ, producing distinct clinical and pathological outcomes. In all nine diseases, CAG repeats become pathogenic only when they exceed a disorder-specific, though broadly comparable, threshold [2]. Full penetrance for HD occurs for a polyQ stretch of *Q* ≥ 39 and 35 < *Q* < 39 may lead to late-onset disease, while *Q* ≤ 35 is considered non-pathogenic. There is a well-established inverse relationship between CAG-repeat length and age of onset: larger expansions drive earlier disease emergence and more severe phenotypes [3]. The CAG repeat and an adjacent CCG triplet encoding a poly-proline (polyP) tract are intrinsically unstable, exhibiting somatic variation across and within brain regions, which may contribute to heterogeneity in onset and symptom progression [4,5]. Clinically, HD typically begins with psychiatric and cognitive disturbances, progresses to pronounced motor deficits and culminates in death within 15–20 years of the onset of the first symptoms. Neuropathologically, the disease is characterized by degeneration of the basal ganglia, driven primarily by the loss of medium spiny neurons in the striatum [6].

## 2. Properties of the huntingtin protein

HTT is a large, ubiquitously expressed protein of more than 3000 amino acids. HTT has a broad functional repertoire including transcriptional and translational regulation, synaptic signalling, vesicular trafficking and macroautophagy. Its extensive interactome further suggests a general scaffolding capacity [7]. Alternative splicing and caspase-mediated cleavage generate smaller HTT fragments. The N-terminal first exon (HTTExon1) contains the pathogenic polyQ expansion [8,9]. HTTExon1 drives formation of the characteristic amyloid aggregates observed in both the cytoplasm and nucleus of neurons in HD patients [10–12], and it readily forms amyloid fibrils *in vitro* [13]. HTT may exert toxicity through gain-of-function and/or loss-of-function mechanisms. Intermediate oligomeric species along the aggregation pathway of HTT have also been implicated in pathogenicity, although their proteotoxic mechanisms are not yet well-defined [14,15].

Amyloid fibril formation is driven by the expanded polyQ tract that is flanked by two distinct domains, an N-terminal sequence of 17 amino acids (N17) and a C-terminal proline-rich domain (PRD) of about 50 amino acids. The highly conserved N17 region forms an amphipathic *α*-helix that can engage with lipid membranes. The N17 is subject to multiple post-translational modifications (e.g. phosphorylation, sumoylation, ubiquitination), that in turn regulates HTT function, subcellular localization, aggregation propensity and proteotoxicity [16–18]. The C-terminal PRD adopts a left-handed type II polyproline helix (PPII) [19] and has been associated with delayed fibrillization and potential protective effects [20,21]. The PRD comprises two pure polyproline tracts (P1 and P2), separated by a proline/glutamine-rich region. Proline's cyclic side chain confers unique properties: extended polyP segments restrict conformational freedom and increase structural rigidity [22], yet they also provide accessible hydrophobic surfaces and hydrogen-bonding capabilities that enable fast, tuneable protein–protein interactions relevant to transcriptional regulation, cell motility and signal transduction [23,24]. While individual prolines are frequently found within intrinsically disordered regions to support flexibility and functionality, natural selection has also positioned polyP tracts at the periphery of aggregation-prone sequences to limit aberrant self-association, effectively serving as gatekeepers [25].

Both the N17 and PRD flanking regions affect the folding and aggregation propensity of the polyQ core [26–28].

## 3. Mechanism of HTTExon1 aggregation

One possible mechanism of aggregation is the classical nucleated growth model, in which formation of a critical nucleus triggers aggregation that then proceeds with a sequential incorporation of monomers into the elongating fibril [29–31]. An alternative model suggests that amyloid nuclei arise first through intermediate higher-order assemblies, such as oligomers [32]. This view is supported by *in vitro* biophysical studies, demonstrating that oligomeric species emerge during aggregation reactions before the appearance of mature fibrils [33,34]. Recently, it was shown that HTT can form reversible liquid-like assemblies that can convert into solid-like assemblies that eventually form amyloid fibrils. The phase transition is regulated by the pathogenic polyQ stretch as well as by the polyQ-flanking polyP domain [35]. Thus, soluble HTTExon1 may first undergo liquid–liquid phase separation to form condensates, that is driven by increasing concentrations of HTTExon1 and an expansion of the polyQ region. The condensates are stabilized by weak hydrophobic interactions between the polyQ region and the PRD and solidify with time to give rise to amyloid fibrils. The HTT folding landscape starting from a HTTExon1 monomer to ultimately forming amyloid fibrils is summarized in figure 1 and outlines possible conformational states that HTTExon1 may adopt (e.g. oligomers, condensates, protofibrils) on its path to amyloid fibril formation. Amyloid fibrils themselves are also not inert and can change over time and depend on the conditions during self-assembly [36]. Each of the transitions between different conformational states of HTTExon1 may be regulated by molecular chaperones and will be discussed in the sections below.

**Figure 1.**
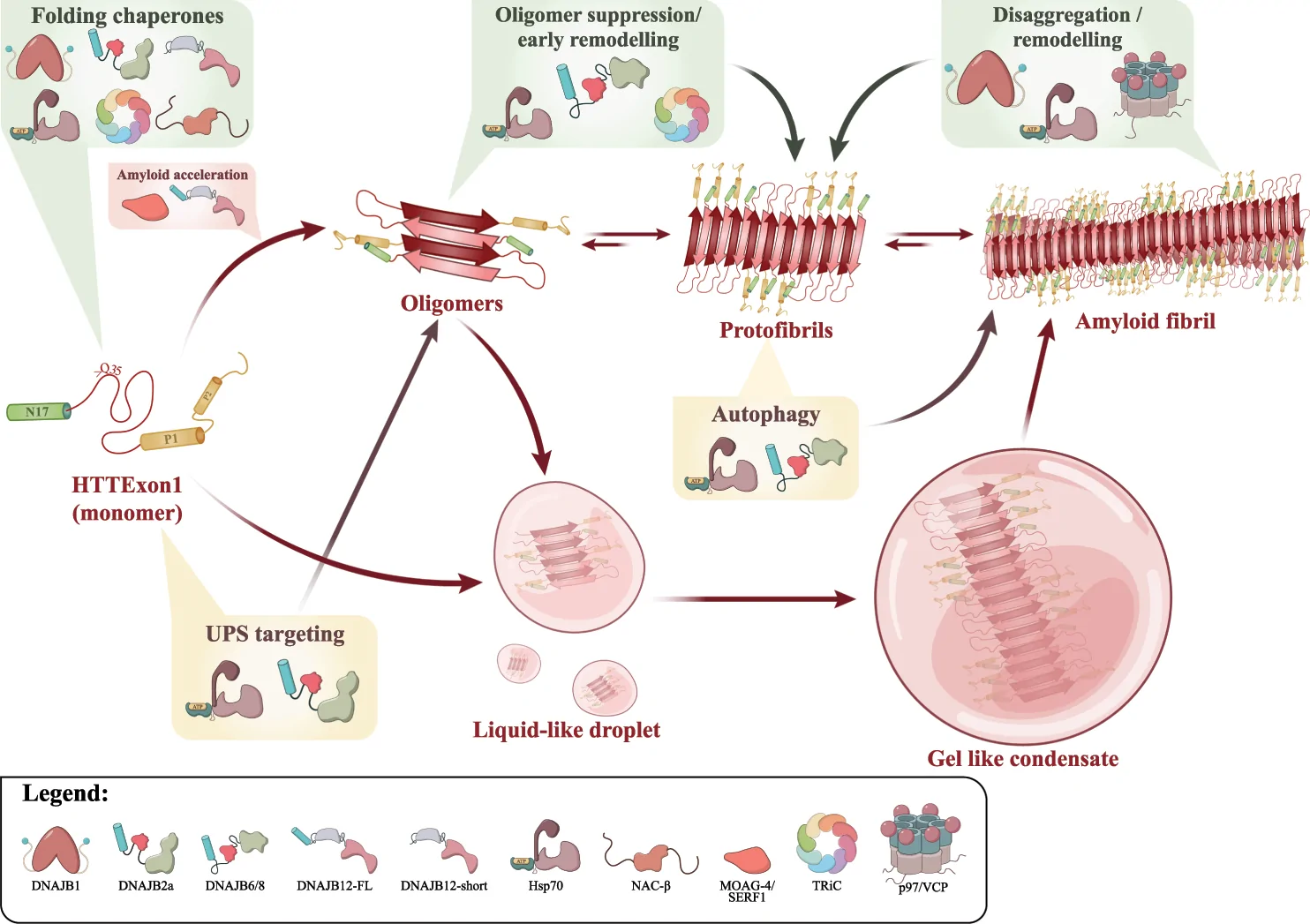
Folding landscape of HTTExon1. HTTExon1 monomers can follow distinct aggregation pathways. In the classical route, soluble monomers self-associate into oligomers, which elongate into protofibrils and eventually mature into highly ordered amyloid fibrils. HTTExon1 can also undergo liquid–liquid phase separation to form dynamic liquid-like droplets that gradually lose mobility and convert into gel-like condensates. These condensates can further transition into amyloid fibrils, highlighting the multifaceted nature of HTTExon1 aggregation. It is not yet established which folding pathway leads to the formation of HTT fibrils and if those paths may exit in parallel. The ability of specific chaperones and chaperone complexes to interact with specific HTT folding entities and to regulate a specific transition in the folding landscape is indicated.

## 4. Molecular chaperones are essential components of the proteostasis network

To support correct protein folding, assembly and targeting as well as to prevent or reverse misfolding, cells rely on an extensive network of molecular chaperones [37]. Traditionally, chaperones have been considered as heat shock proteins (Hsps), named for the stress response that originally revealed them [38]. However, it is now understood that most chaperones can also be expressed constitutively or are induced in response to very diverse stimuli [39,40]. They are further subdivided by their molecular weight into major ATP-dependent families, including Hsp60s, Hsp70s, Hsp90s, Hsp100s and ATP-independent chaperones such as small Hsps and J-domain proteins (JDPs), which enhance specificity in substrate recognition and delivery to ATP-dependent chaperones [41–43].

Molecular chaperones regulate all steps of the lifespan of a protein [43]. Chaperones already engage with the nascent polypeptide chain as it emerges from the ribosome and aid in their subcellular targeting and folding. Through coordinated action of chaperones and co-chaperones, the substrate protein is bound and released using iterative ATP-driven cycles, guiding the protein toward its correct conformation [44]. Chaperones also act on misfolded proteins and promote their refolding to suppress the formation of protein aggregates. The formation of protein aggregates is however not a dead-end point as they can be disaggregated by molecular chaperones. Even amyloid fibrils that are composed of rigid *β*-sheet-containing elements can be resolubilized by various chaperone complexes [45–48].

However, in aging or disease contexts, large aggregates can sequester substantial fractions of the chaperone network, the associated degradation machinery and many other endogenous proteins, causing a loss of function that may contribute to disease pathology. Such sequestration compromises the cell's ability to neutralize aberrantly folded proteins and diminishes its capacity to respond to additional proteotoxic stress, ultimately contributing to an exhaustion of the proteostasis network and cell death [49]. Given their broad functional versatility and central role in protein quality control, chaperones represent highly promising therapeutic targets for neurodegenerative diseases [50,51].

## 5. Regulation of the HTTExon1 folding landscape by molecular chaperones

Molecular chaperones have been implicated in suppressing, delaying and even reversing the aggregation of mutant HTT. For example, modulation of the expression of Hsp70, JDPs, Hsp110 or TRiC (T-complex protein ring complex) affected the aggregation or toxicity of HTT in cultured cells, flies, nematodes and HD mouse models [47,52–57]. *In vitro* analyses have shown that HTT aggregation can be suppressed by *β*NAC (beta subunit of the nascent polypeptide-associated complex), the JDP DNAJB6, TRiC and the trimeric chaperone complex composed of Hsp70, DNAJB1 and the nucleotide exchange factor Apg2 [47,52,54, 58–60]. We summarize below our current knowledge of the regulation of the HTT folding landscape by the individual chaperone classes. Their involvement in the regulation of the different folding entities of HTT (e.g. the interaction with and suppression of the aggregation of soluble HTTExon1 or targeting of HTTExon1 oligomers to the proteasome) is depicted in figure 1. The domain architectures of the chaperones that can regulate HTT are shown in figure 2, which summarizes our knowledge on the diversity of HTT-regulating chaperones. Figure 3 provides an overview of the interaction sites of the different chaperones within HTTExon1 that have already been mapped.

**Figure 2.**
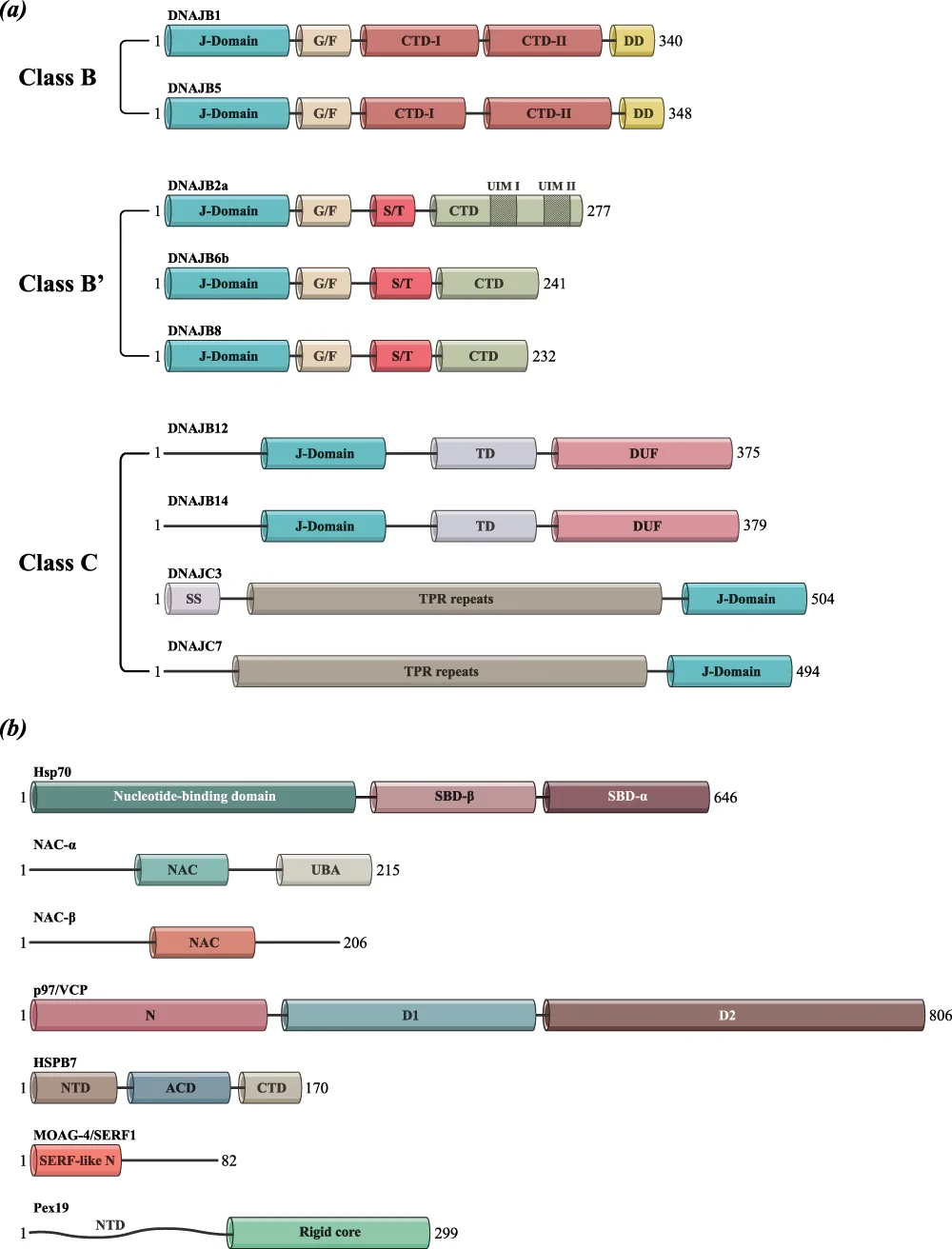
Domain organization of chaperones that interact with and remodel HTTExon1. (*a*) Schematic representation of representative J-domain proteins (JDPs) from Classes B/B′ and C that interact with HTTExon1: Class B (top), Class B′ (middle) and Class C (bottom). The J-domain, a characteristic feature of all JDPs, is shown in teal across all members. The G/F linker is highlighted in light beige, while C-terminal domains (CTDs) are colour-coded by class: red-brown for Class B and pale green for Class B′. Additional class-specific features are individually colour-coded to reflect their structural and functional diversity. CTD, C-terminal domain; DD, dimerization domain; DUF, domain of unknown function; SS, signal sequence; ST, Ser-/Thr-rich region; TD, transmembrane domain; TPR, tetratricopeptide repeat; UIM, ubiquitin-interacting motif. (*b*) Schematic representation of co-chaperones Hsp70, *α*NAC, *β*NAC, p97/VCP (valosin-containing protein), the small heat shock protein HSPB7 and MOAG-4/SERF1 (modifier of aggregation) and the peroxine Pex19. Each protein is colour-coded according to its distinct domain architecture. Domain abbreviations: ACD, *α*-crystalline domain; CTD, C-terminal domain; D1, D2, ATPase domains; NAC, nascent polypeptide-associated domain; NBD, nucleotide-binding domain; NTD, N-terminal domain; SBD-*α*-/*β*, C-terminal substrate-binding domains; SERF-like N, small EDRK-rich factor 1/2-like, N-terminal; UBA, ubiquitin-associated domain.

**Figure 3.**
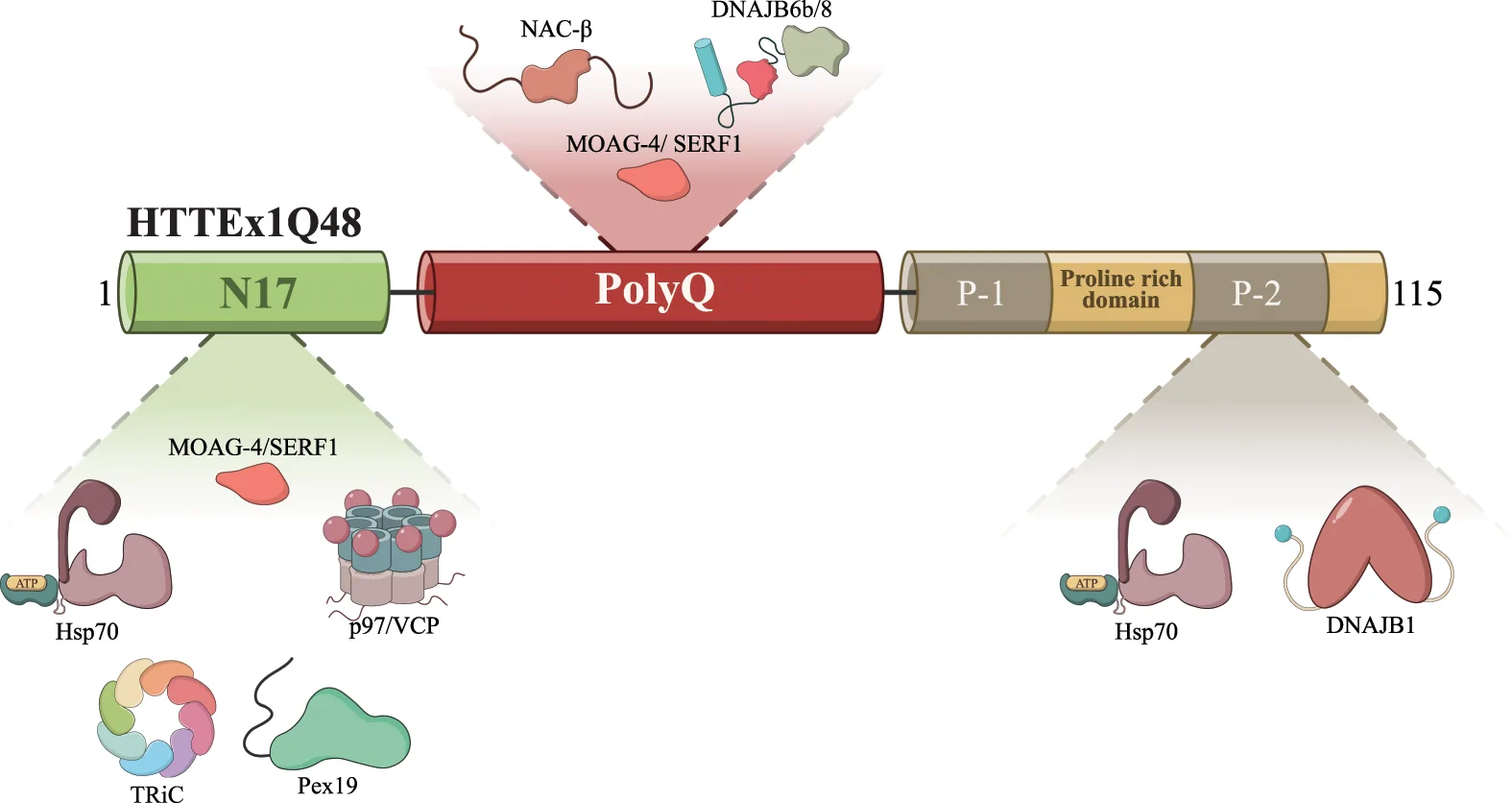
Domain architecture of HTTExon1 and mapped chaperone interactions. Schematic representation of HTTExon1 highlighting its domain architecture and the spatial positioning of chaperones known to interact with specific regions. HTTExon1 comprises the N17 domain (green), a central polyglutamine (polyQ) stretch (red) and a proline-rich domain (PRD) containing two polyproline segments, P1 and P2, shown in tan. Chaperones are positioned beneath their reported binding sites of HTTExon1. Hsp70, p97/VCP, TRiC and MOAG-4/SERF1 associate with the N17 domain. *β*NAC, DNAJB6b/8 and MOAG-4/SERF1 interact with the polyQ region. The PRD is recognized by DNAJB1 and Hsp70.

### (a) J-domain proteins

Based on domain composition, JDPs are grouped into different classes (figure 2*a*) [61]. Only Classes B and C JDPs have been shown to interact with and remodel HTT. Class B JDPs are divided into canonical and non-canonical (Class B′) types. Canonical Class B proteins have a C-terminal substrate-binding and dimerization domain, whereas B′ JDPs lack these features and instead contain a serine/threonine-rich region followed by a substrate-binding domain immediately after the G/F segment. Class B members that can regulate HTT folding are DNAJB1 and DNAJB5, whereas DNAJB2a, DNAJB6 and DNAJB8 belong to Class B′ (figure 2*a*). Class C JDPs represent the largest and most diverse group of JDPs. Although they contain a J-domain, its position is variable and their domain architectures are highly diverse, including ubiquitin-binding domains, cysteine-rich regions, GTP-binding domains, tetratricopeptide repeats (TPRs) and clathrin-binding domains, consistent with broad functional roles. Members of this class that regulate HTT are DNAJB12, DNAJB14 and DNAJC3, and DNAJC7 (figure 2*a*). DNAJB12 and DNAJB14 have only very recently been re-assigned to Class C [62].

#### (i) DNAJB1

A trimeric chaperone complex consisting of Hsp70, Hsp110 and DNAJB1 can suppress the amyloid fibril formation of HTT Exon1Q_48_ and can also disaggregate preformed HTTExon1Q_48_ fibrils. Depletion of these chaperones in HD patient-derived neural progenitor cells (NPCs) led to an increase in HTTQ_44_ aggregation [47]. Among the three chaperones, the JDPs emerged as the most concentration-sensitive factor *in vitro* and exerted the strongest effect on HTTQ_44_ aggregation upon knockdown in NPCs. In line with this observation, overexpression of DNAJB1 significantly reduced the aggregation of HTTExon1Q_97_ in cultured HEK cells [47]. Subsequent studies revealed that HTTExon1 interacts with DNAJB1 (and Hsp70) through its PRD [58]. Mapping studies using cross-linking mass spectrometry localized the HTT-binding site within DNAJB1 to a nine-amino-acid segment in the hinge region between C-terminal domains I and II. Notably, a histidine residue within this region is highly conserved across related JDPs. Substitution of this histidine with alanine restored HTTExon1 fibril formation, while leaving the handling of other chaperone substrates unaffected. These findings led to two conclusions: His244 of DNAJB1 is essential for suppressing HTTExon1 aggregation by the chaperone complex, and its function is highly specific to HTT.

To elucidate the role of His244, molecular dynamics simulations and docking studies were conducted. These in silico analyses indicated that His244 forms a hydrogen bond with a glutamate residue in the hinge region of DNAJB1. This interaction, together with additional contacts identified in the simulations, probably stabilizes the DNAJB1–HTTExon1 interface and thereby inhibits aggregation [58]. A remaining paradox was that His244 is also conserved in other JDPs, such as DNAJA1, which do not exhibit chaperone activity toward HTT. This suggested that additional structural or functional features of the DNAJB1 contribute to HTT recognition. Comparative analysis of DNAJA1 and DNAJB1 revealed two major differences: first, DNAJB1 possesses a longer linker connecting the J-domain to the C-terminus; second, DNAJA1 contains a zinc finger-like region (ZFLR) that is absent in DNAJB1. Domain deletion and swapping experiments demonstrated that replacing the shorter DNAJA1 linker with the longer DNAJB1 linker partially conferred suppression of HTT aggregation activity. The resulting chimeric protein was able to significantly delay, though not fully prevent, HTT aggregation in the presence of Hsp70 and Apg2. These results imply that the additional elements within DNAJB1 contribute to HTT binding and possibly to its transfer to Hsp70 during the chaperone cycle [58].

The interaction with HTT fibrils and their subsequent disaggregation was also examined. Structural studies of HTTExon1 fibrils showed that the PRD is positioned outside the fibril core, rendering it accessible to chaperones [63]. HTT fibrils lacking the identified proline stretch were no longer recognized by DNAJB1 and consequently failed to get disaggregated by the chaperone complex. Mutation of His244 reduced DNAJB1 affinity for HTT fibrils and consequently markedly impaired the ability of the trimeric chaperone system to disaggregate them. However, unlike its effect on soluble HTT, the His244 mutation did not completely abolish activity toward fibrils. Together, these findings indicate that suppression of soluble HTT aggregation and disassembly of HTT fibrils rely on overlapping binding determinants within DNAJB1, including His244, while also requiring additional, as yet unidentified, interaction sites or downstream steps involved in HTT transfer to Hsp70 [58].

#### (ii) DNAJB2a

The primary role of DNAJB2 is to facilitate the degradation of Hsp70 substrate proteins via the ubiquitin–proteasome system (UPS). DNAJB2 promotes the ubiquitylation of protein substrates by the E3 ligase CHIP (carboxy-terminus of Hsc70-interacting protein) and, by binding to the polyubiquitin chains, it protects them from deubiquitylation to facilitate the subsequent degradation by the proteasome [64]. DNAJB2a can target HTT to the proteasome for degradation in a manner dependent on its HPD (His-Pro-Asp sequence required for Hsp70 binding) and ubiquitin interaction motifs, further supporting that DNAJB2a cooperates with Hsp70 and CHIP [64–67].

#### (iii) DNAJB5 and DNAJC3

Using a genetic screen of *Drosophila* JDPs, CG5001 and P58IPK were identified to reduce HTTExon1Q_103_ aggregation in yeast [68]. CG5001 is a homolog of human DNAJB5 and P581PK is a homolog of human ER (endoplasmatic reticulum)-residing DNAJC3. Both JDPs are involved in ER stress regulation [69]. Their ability to ameliorate HTTExon1Q103 aggregation has been validated in *Drosophila* and in a *Drosophila* S2 cell line [68].

#### (iv) DNAJB6

In a targeted screen for modifiers of polyQ aggregation, two closely related non-canonical Class B JDPs, DNAJB6 and DNAJB8, were identified as polyQ suppressors [70]. DNAJB8, predominantly expressed in the testis, and DNAJB6, which is expressed across multiple tissues including the brain, act as highly effective suppressors of polyQ aggregation *in vitro* and in cellular models [70–72]. Mechanistic analyses revealed that DNAJB6 can suppress the aggregation of polyQ by inhibiting its primary nucleation [52] and can also affect secondary nucleation of amyloid growth [72]. DNAJB6 uses its serine/threonine-rich domain, which presents multiple surface-exposed hydroxyl groups, to interact with polyQ peptides. This interaction interferes with the hydrogen bonding required for amyloid fibril stability and thereby inhibits primary nucleation.

Amyloid aggregates are largely inert to autophagic or proteasomal degradation [73]. It has recently been demonstrated that fragmentation of amyloid fibrils by molecular chaperones into smaller particles enables degradation by autophagy [74]. Efficient clearance of protein aggregates was shown to depend on a fragmentation of protein aggregates by a chaperone complex in HTT in conjunction with the proteasomal regulatory 19S particle, a process that enables the recruitment and clustering of selective autophagy receptors necessary to initiate phagophore formation. An additional role was attributed to DNAJB6, which was shown to pair up with Hsp70 and Hsp110 and the 19S regulatory particle (RP) of the proteasome to break down HTT aggregates into smaller particles that could then be degraded by autophagy. Acting in concert, these components not only drive the fragmentation of protein aggregates, but also promote their compaction, thereby facilitating the clustering of selective autophagy receptors and the localized formation of phagophores. These observations suggest that this fragmentase activity could be broadly required for aggrephagic clearance of different aggregate types, including pathogenic HTT aggregates [74].

The disaggregation activity of the DNAJB6/Hsp70/Hsp110 complex probably differs from DNAJB1/Hsp70/Hsp110 [46,47,75] as its fragmentation activity requires the 19S RP and the products of the fragmentation are targeted for autophagic degradation. Thus, this chaperone autophagy pathway seems protective as it eliminates aggregates and might not generate seeding competent species by disaggregating amyloid fibrils, as shown for the DNAJB1/Hsp70/Hsp110 chaperone complex [76]. However, the observation that autophagy preferentially degrades non-fibrillar polyQ aggregates, raises the question of whether DNAJB6/Hsp70/Hsp110/19S indeed acts on large amyloid fibrils or rather on smaller assemblies of the amyloid protein [74,77].

#### (v) DNAJB12 and DNAJB14

A cellular screen for modulators of polyQ aggregation identified DNAJB12 as an additional regulator of HTTExon1Q_134_ aggregation [78]. Interestingly, expression of different isoforms of DNAJB12 showed opposing behaviour. While the ectopic expression of DNAJB12-short suppressed HTT-polyQ aggregation, expression of full-length DNAJB12 (DNAJB12-FL) aggravated HTTQn aggregation. Thus, the diverse functional spectrum of JDPs could be further enhanced by their different isoforms, which can also give rise to a variety of different and thus potentially specialized heterocomplexes as shown for DNAJB12-FL–DNAJB14-FL, indicating a role for DNAJB14 in HTT regulation as well [78]. Both JDPs are anchored into the ER membrane and have domains that are located in the ER lumen as well as in the cytosol. Their sub-cellular localization could change for the different isoforms, so that they could be able to interact with cytosolic HTT [78].

#### (vi) DNAJC7

A very recent cellular CRISPRi knockdown screen identified DNAJC7 as a suppressor of HTTExon1Q_72_ aggregation, which has so far only been shown to be involved in the regulation of Tau and TDP-43 [57]. Cellular studies could further show that overexpression of DNAJC7 leads to a reduction in HTTExon1Q_72_ aggregates and that DNAJC7 co-localizes with HTTExon1Q_72_ aggregates in cells. It is not known yet which steps in the HTT folding landscape are regulated by DNAJC7.

### (b) Heat shock protein 70

JDPs can inhibit the self-assembly of polyglutamine proteins into amyloid-like fibrils either alone (e.g. DNAJB6/8 [52]) or together with Hsp70 [79,80]. A combination of complementary approaches was used to map the interaction between Hsp70 and HTTExon1. Hsp70 associates with the 17-residue N-terminal region of HTTExon1, as evidenced by the observation that its effect on aggregation is independent of polyQ length but requires the presence of the N-terminal flanking sequence of the polyQ tract [56].

### (c) p97/VCP

HTTExon1Q_97_ fibrils can also be disaggregated by the AAA+ (ATPases associated with diverse cellular activities) protein p97 [81]. AAA+ proteins form hexameric structures and use ATP hydrolysis to thread aggregated proteins through the inner channel to mechanically disentangle aggregated proteins [82]. p97 recognizes the N17 domain of HTTExon1 fibrils [81].

### (d) NAC

The heterodimeric NAC chaperone complex, that consists of *α*NAC and *β*NAC subunits, has been well characterized for its role in assisting the folding of newly synthesized proteins that emerge from the ribosomal exit tunnel. However, NAC can also dissociate from the ribosome and can bind to aggregated proteins [83]. The *β* subunit of NAC can bind with its positively charged N-terminus to aggregation-prone proteins such as the polyQ tract of HTTExon1 [59]. This binding leads to a suppression of the aggregation propensity of HTTExon1 *in vitro* and could also exert a rescue effect *in vivo*. Notably, the N-terminus of *β*NAC alone was sufficient for the suppression of HTTExon1 aggregation, which is exactly the binding site for the association with the ribosome, suggesting that these interactions are mutually exclusive. Thus, NAC exerts ribosome-dependent chaperone functions to assist in the folding of nascent chains upon their synthesis and ribosome-independent chaperone functions to act as a holdase to suppress the aggregation of, e.g. HTTExon1 [59,84].

### (e) TRiC

TRiC belongs to the group II chaperonins that form a ring-shaped, hetero-oligomeric chaperone complex to promote protein folding through iterative cycles of ATP binding and hydrolysis [85]. The chaperone complex consists of two stacked rings with eight subunits, each (CCT1–CCT8) forming a central cavity. The different subunits allow the interaction with different protein substrates [86]. Recent studies have shown that its individual subunits exhibit distinct substrate-recognition specificities. Subunit 1 and to a lesser extent also subunit 4, for instance, recognize HTTExon1 and suppress its aggregation [54,60]. Using photo-cross-linking, it was shown that TRiC inhibits the aggregation of HTTExon1 by binding hydrophobic amino acids of the N17 domain [87].

### (f) HSPB7

Among the small Hsps (sHsps), HSPB7 showed the strongest effect on polyQ proteins in an HD model. HSPB7 suppressed polyQ aggregation and exerted its protective effect independently of Hsp70 and the proteasome, but relied on an active autophagy pathway, suggesting that HSPB7 suppresses polyQ toxicity through a non-canonical mechanism [88].

### (g) MOAG-4/SERF1

A chemical mutagenesis screen in a *Caenorhabditis elegans* polyQ model identified MOAG-4 as a modulator of polyQ, but also of *α*-synuclein and A *β* aggregation [89,90]. The human ortholog is SERF1, an RNA-organizing protein enriched in positively charged amino acids. Structural analyses could show that SERF1 binds to the N17 domain of HTTExon1 [91]. Interestingly, SERF1 promotes HTTExon1 aggregation [92]. This poses the question whether SERF1 is actually a chaperone. However, a selective protein aggregation-promoting activity was also shown for sHsps, e.g. YocM, Hsp42 and HSP-17 [93–95], and it is thus possible that this aggregase/sequestrase activity of SERF1 is physiologically relevant and could even be cytoprotective in response to specific proteotoxic challenges and may also cooperate with the proteostasis network.

### (h) Engineering chaperones

One proposed strategy for counteracting HTT aggregation is to engineer molecular chaperones to boost chaperone capacity of affected cells. Such a strategy has already been used for Hsp104 to mitigate the proteotoxic effects of TDP-43, FUS and *α*-synuclein in models of amyotrophic lateral sclerosis (ALS) and Parkinson's disease [96–98]. However, many chaperones, including Hsp104, depend on ATP that may present challenges for therapeutic development. In parallel, there are several ATP-independent human disaggregases, including high-temperature requirement A1 (HTRA1) protease and PEX19 that act as chaperone and as an import receptor for peroxisomal membrane proteins [99]. Both have been shown to reverse the formation of *α*-synuclein and HTTExon1 aggregates [100,101]. The engineering of the PEX19-based chaperones started with a yeast toxicity-based screen and identified two yeast PEX19 variants, scPEX19-FV (L288F/E292V) and scPEX19-FI (L288F/E292I), that could both rescue the toxicity of mutant HTTExon1 in yeast. These mutations map to the *α*4 helix of PEX19 that is highly conserved and has thus been used to generate the human variants hsPEX19-FV (M255F/Q259V) and hsPEX19-FI (M255F/Q259I), which also suppressed mutant HTTExon1 aggregation *in vitro* and in mammalian cells and rescued HTT-induced neurite degeneration in primary striatal neurons. Mechanistically, the engineered PEX bound to the N17 domain of HTTExon1 and thereby delayed its aggregation [100]. Collectively, these observations highlight ATP-independent chaperones as promising candidates for therapeutic intervention in neurodegenerative diseases.

#### (i) Targeting huntingtin with chemical chaperones and antibodies

Chemical chaperones are low-molecular-weight compounds and can be grouped into two main categories: osmolytes and hydrophobic compounds. They act in a largely nonspecific manner. Chemical chaperones typically exert their effects only at high concentrations, which has limited their consideration as therapeutic agents. Nevertheless, renewed interest has emerged for some of these compounds as potential treatments for neurodegenerative diseases. This is owing to their complex mechanisms of action that may affect multiple stages of the neuropathological cascade of amyloid proteins such as HTT. *In vitro* screenings revealed that several disaccharides are capable of inhibiting polyglutamine-mediated protein aggregation *in vitro* and enhanced cell viability in a cellular model of Huntington disease [102,103]. Among these compounds, trehalose was the most effective. Oral administration reduced polyglutamine aggregates in the cerebrum and liver, ameliorated motor impairments and extended lifespan in a transgenic mouse model of HD. These beneficial effects are proposed to arise from direct binding of trehalose to expanded polyglutamine tracts, resulting in stabilization of partially unfolded polyglutamine-containing proteins [102]. Another example is the short-chain fatty acid sodium 4-phenylbutyrate (PBA). It could be demonstrated that intraperitoneal injections of PBA significantly extended survival and ameliorated neurodegeneration in an HD mouse model [104]. Very recently, it could be shown that the polyphenol curcumin can delay HTTExon1Q_44_ fibril formation *in vitro* by inhibiting the primary and secondary nucleation [105]. HTT fibrils that were ultimately formed in the presence of curcumin displayed an altered polyQ core structure. Treatment with curcumin reduced HTT pathology in a cellular model that could be attributed to the delayed aggregation observed *in vitro* or to the altered properties of the fibrils that are formed in the presence of curcumin [105]. Of note, amyloid dyes such as Congo red, thioflavine S, chrysaine G and direct yellow 20, that directly bind to *β*-sheet structures and are for that purpose used to study fibril formation of amyloid proteins, were also shown to inhibit the formation of HTT fibrils *in vitro* and for Congo red also in cells [106].

Engineered intracellular antibodies (intrabodies) have also been proposed as a therapeutic strategy to ameliorate HD pathology. Intrabodies are single-chain and single-domain variable fragments that can retain the affinity and specificity of full-length antibodies, but can be easily cloned and engineered as genes. Several intrabodies that selectively bind mutant HTT, have been tested and those that target the polyglutamine flanking region showed a therapeutic benefit in several model systems [107]. These findings support intrabodies as a promising strategy for treating protein-misfolding neurodegenerative diseases.

## 6. Conclusions and outlook

Several chaperones and chaperone complexes can bind to HTT and regulate its folding landscape. Chaperones use different domains for their interaction with HTT and also bind to distinct sites within HTTExon1, suggesting different mechanisms of HTTExon1 aggregation suppression. For example, the amphipathic N17 domain is bound by Hsp70, TRiC, PEX, MOAG-4/SERF1 and p97/VCP that thus target and neutralize the domain that initiates the aggregation of HTT. The polyQ stretch is bound by DNAJB6/8, *β*NAC and MOAG-4/SERF1 using, e.g. hydrogen bonds and electrostatic interactions thereby directly interfering with the aggregation process. The C-terminal polyQ-flanking PRD that can buffer the aggregation propensity of the polyQ domain is bound by DNAJB1/Hsp70 (figure 3). The versatile binding modes could be even higher when taking into account the different conformational states of HTT. While most studies focused on the interaction of chaperones with soluble HTT, some chaperones are also capable of interacting with aggregated HTT (e.g. DNAJB1/Hsp70/Hsp110, p97) and probably also additional assembly states (oligomers, condensates, protofibrils; figure 1).

Targeting molecular chaperones represents a promising but also challenging therapeutic strategy for HD. To date, only a limited number of chaperone-directed compounds have been developed, and even fewer have progressed to clinical testing. The complexity and modularity of chaperone-mediated protein folding, refolding, disaggregation and degradation pathways offer numerous opportunities for therapeutic intervention. The observation that chaperones cannot only suppress aggregation but also disaggregate aggregated proteins that then liberate seeding-competent protein conformations calls for caution when modulating chaperone levels or their activity. In addition, any perturbation of chaperone levels/activities could lead to compensatory effects by other chaperones and proteases. For example, overexpression of Hsp110 increased the burden of amyloid aggregates and caused cellular and organismal toxicity [108]. Mechanistically, Hsp110 is an essential component of the disaggregating chaperone complex whose activity could release seeding-competent protein species and in turn increase aggregation and propagation [45,47,76]. Moreover, Hsp110 was shown to be a negative regulator of autophagy. Overexpression of Hsp110 thus severely compromised the cell's ability to degrade protein aggregates, exacerbating the protein aggregation load [108]. Thus, the activity of chaperones needs to be precisely controlled and their expression/activation spatio-temporally restricted.

## Data Availability

This article has no additional data.

## Theme

One contribution of 20 to a Theo Murphy meeting issue ‘ProteostaSys: a systems view of proteostasis'.
